# *DHQS* regulates pollen-pistil interaction via the shikimic acid pathway in *Arabidopsis thaliana*

**DOI:** 10.1080/15592324.2026.2670874

**Published:** 2026-05-29

**Authors:** Xiu Wang, Xiongbo Peng

**Affiliations:** a State Key Laboratory of Hybrid Rice, College of Life Sciences, Wuhan University, Wuhan, People's Republic of China; b Anhui Province Key Laboratory of Crop Integrated Pest Management, Anhui Agricultural University, Hefei, People's Republic of China

**Keywords:** Shikimic acid, pollen, pollen-pistil interactions, DHQS

## Abstract

The normal production of pollen and proper pollen-pistil interaction are critical for double fertilization in *Arabidopsis*. Previous studies have demonstrated that DHQS affects female gametophyte development and pollen tube funicular guidance during fertilization. However, whether DHQS influences pollen development and pollen-pistil interaction remains unknown. In this study, we analyzed pollen and sperm cell morphology in *dhqs-1*/+ mutant. Results showed that DHQS does not affect sperm cell formation but significantly reduces pollen grain size. Pollen germination assays revealed no impairment in *dhqs-1* pollen germination. However, semi-in vitro experiments demonstrated that *dhqs-1* pollen tubes exhibited delayed growth through stigmas compared to wild-type. Notably, supplementation of shikimic acid in the medium significantly alleviated the growth inhibition of *dhqs-1* pollen tubes in wild-type stylar tissue, underscoring the essential role of shikimic acid in mediating pollen-pistil interaction. These findings suggest that DHQS regulates pollen-pistil interaction through the shikimic acid pathway, providing novel insights into the molecular mechanisms underlying fertilization in plants.

## Introduction

Sexual reproduction in angiosperms is a complex and highly coordinated process that culminates in double fertilization, a unique event essential for seed formation.^1^ This process relies on the successful production of functional male gametophytes (pollen) and their precise interaction with female tissues, collectively known as pollen-pistil interaction. Following pollination, the pollen grain hydrates, germinates, and produces a pollen tube that must navigate through the stigma and transmitting tract to deliver the sperm cells to the ovule.^2^ Defects at any stage, from pollen development to pollen tube guidance, can lead to reproductive failure.

Compounds that regulate pollen tube growth mainly include flavonoids, sphingolipids, reactive oxygen species (ROS), calcium ions (Ca²⁺), and phytohormones. These molecules function through complex signaling networks to coordinately maintain the polar growth and integrity of the pollen tube.^3^ In rice, flavonoid and triterpenoid metabolism modulate *α*-amylase activity to maintain pollen tube growth on the stigma.^4^ During oscillatory growth, sphingolipid-mediated signaling pathways act in concert with Ca²⁺ gradients to maintain cell wall elasticity and membrane integrity.^5^ ROS function as signaling molecules and play important roles in pollen–stigma recognition.^6^ Meanwhile, Ca²⁺ signals, together with RAC/ROP small GTPases and the actin cytoskeleton, form feedback circuits that regulate the direction and rate of pollen tube growth.^7^ ^,^ ^8^ Gibberellin (GA) biosynthesis in pollen tubes is essential for their growth, and GA deficiency inhibits both germination and elongation.^9^ Low-molecular-weight factors derived from pistils are also critical for pollen germination and tube growth. These include brassinosteroids,^10^ sulfonated azadecalins,^11^ the amino acid D-serine, which influences pollen tube growth through the regulation of glutamate receptor-like Ca²⁺ channels,^12^ and *γ*-aminobutyric acid (GABA).^13^

The shikimate pathway is a crucial metabolic route in plants, linking primary and secondary metabolism by synthesizing aromatic amino acids and a wide array of downstream compounds.^14^ Notably, the growing pollen tube possesses a functional shikimate pathway for aromatic amino acid synthesis.^15^ In *Arabidopsis thaliana*, 3-dehydroquinate synthase (DHQS), which catalyzes the second step of the shikimate pathway, is encoded by a single-copy gene. Previous research has established that DHQS is not only vital for general plant development but also plays specific roles during reproduction.^16^ For instance, disruption of DHQS function has been shown to affect female gametophyte development and, more specifically, impair pollen tube funicular guidance, a critical step for successful fertilization.^16^ Despite these findings, the role of DHQS in the earlier, equally critical stages of male reproduction-specifically pollen development and the initial pollen-pistil interaction-has remained unexplored.

In this study, we investigated the potential functions of DHQS in male gametophyte development and function using the heterozygous *dhqs-1*/+ heterozygous mutant line. We analyzed pollen morphology and sperm cell formation and assessed pollen germination efficiency. Furthermore, we examined the dynamics of pollen tube growth during its passage through the pistil. Our results demonstrate that while DHQS is dispensable for sperm cell specification, it is required for normal pollen grain development. Moreover, we uncovered a post-pollination role for DHQS, as *dhqs-1* mutant pollen tubes show a significant delay in growth on the stigma. Importantly, we show that pollen tube growth on the stigma defect is directly linked to shikimic acid availability, as exogenous application of shikimic acid significantly alleviates the mutant phenotype. These findings reveal the novel function for DHQS in mediating pollen-pistil interaction, highlighting the critical role of the shikimate pathway in the early events of fertilization beyond its previously known functions.

## Material and methods

### Plant materials and growth conditions

*A. thaliana* plants were maintained in a growth chamber under a 16-h-light/8-h-dark cycle at 22 °C. The *dhqs-1* allele was isolated with hygromycin resistance from our mutant library based on *quartet1* (*qrt1*) with *Columbia* (*col-0*) background.^17^ ^,^ ^18^

### Microscopy and imaging

Samples were observed under a confocal laser scanning microscope (Leica SP8 CLSM). The following excitation (ex) and emission (em) wavelengths (in nm) were applied: for GFP, ex 488/em 500–535; for RFP, ex 552/em 590–700.

### Semi-*in vitro* pollen tube growth assay

Semi-*in vitro* pollen tube growth assay was performed as previously described.^19^ The basal pollen germination medium consisted of 1 mM CaCl₂, 1 mM Ca(NO₃)₂, 1 mM MgSO₄, 0.01% (w/v) H₃BO₃, and 18% (w/v) sucrose. The pH of the basal medium was adjusted to 7.0, followed by the addition of 1% (w/v) agarose. For the experimental group, 100 mg/L shikimic acid was pre-added to the basal medium, after which the pH was adjusted to 7.0 and 1% (w/v) agarose was added. Flowers at stage 12 on wild-type inflorescences were emasculated and pollinated 12 h later with pollen from *dhqs-1*/+ plants. Thirty minutes after pollination, the stigmas and styles were excised from the pistils and placed on the above-mentioned pollen tube growth medium with or without shikimic acid, then incubated at 22 °C for 3 hours. Observation was performed under a confocal laser scanning microscope. Pollen tubes emitting green fluorescence were identified as *dhqs-1* pollen tubes.

### Quantification and statistical analysis

All statistics were calculated in GraphPad Prism 7. All measured data are presented with sample numbers (*n*) indicated in the Methods and figure legends.

## Result

### 3-Dehydroquinate synthase (DHQS) in the shikimate biosynthesis pathway regulates pollen grain size and does not significantly affect sperm cell development in *Arabidopsis thaliana*

Our previous work demonstrated that loss of DHQS, a key enzyme in the shikimate pathway, disrupts female gametophyte development and thereby impairs funicular guidance of pollen tubes.^16^ To further investigate the role of DHQS in male gametophyte development, we conducted the present study. The T-DNA insertion construct used in our laboratory carries a pollen-specific green fluorescent protein (GFP) marker, allowing unambiguous discrimination between wild-type and *dhqs-1* mutant pollen grains in *dhqs-1*/+ heterozygous plants: pollen grains exhibiting GFP fluorescence represent the homozygous *dhqs-1* mutant genotype.

**Figure 1.**
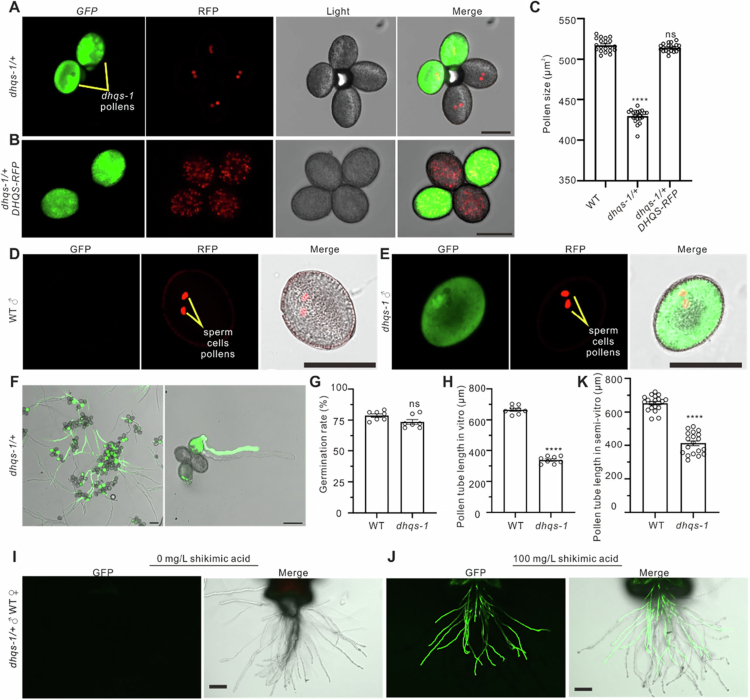
Shikimic acid affects pollen-pistil interactions in Arabidopsis thaliana. A, Pollen grains morphology of wild-type (WT) (lacking GFP fluorescence) and dhqs-1 (exhibiting GFP fluorescence) in dhqs-1/+ plant. B, Pollen morphology of wild-type (WT) and dhqs-1 DHQS-RFP in dhqs-1/+ DHQS-RFP plant. C, Pollen size in WT, dhqs-1 and dhqs-1 DHQS-RFP. The values are means ± SEM from 20 biological replicates. D, Morphology of sperm cells in WT pollen. E, Morphology of sperm cells in dhqs-1 pollen. F, In vitro culture of WT pollen grains (lacking GFP fluorescence) and dhqs-1 pollen grains (exhibiting GFP fluorescence). G, Pollen germination rate (%) in WT and dhqs-1. The values are means ± SEM from 7 biological replicates. H, Pollen tube length in WT and dhqs-1 in vitro. The values are means ± SEM from 8 biological replicates. I, Semi-in vitro pollen tube growth assay following pollination of WT pistils with dhqs-1/+ pollen in 0 mg/L shikimic acid. J, Semi-in vitro pollen tube growth assay following pollination of WT pistils with dhqs-1/+ pollen in 100 mg/L shikimic acid. K, Pollen tube length in WT and dhqs-1 following pollination of WT pistils with dhqs-1/+ pollen in 100 mg/L shikimic acid. The values are means ± SEM from 19 biological replicates. Scale bars: 10 μm (A, B, and E) and 25 μm (I and J). Statistical significance was determined by Student's t-test; ns, not significant; ****P < 0.0001.

Examination of pollen tetrads from *dhqs-1*/+ plants revealed that fluorescent (*dhqs-1*) pollen grains were consistently smaller than their non-fluorescent (wild-type) counterparts (Figure 1A). This pollen grain morphology defect was partially rescued in *dhqs-1* complementation lines (expressing DHQS-RFP), confirming that DHQS is required for normal pollen development (Figure 1B, C). To assess potential defects in sperm cell formation, we introduced a sperm cell‑specific nuclear marker (pHTR10-RFP) into the *dhqs-1*/+ genetic background. Specifically, homozygous pHTR10-RFP plants were crossed with *dhqs-1*/+ plants, and appropriate F2 progeny were selected for analysis. Microscopic examination of pollen from these *dhqs-1*/+ plants expressing pHTR10-RFP showed that sperm cells were still present in *dhqs-1* mutant pollen grains (Figure 1D, E), indicating that DHQS is not required for sperm cell formation in pollen. Based on these observations, we conclude that loss of DHQS function in *A. thaliana* results in reduced pollen grain size during male gametogenesis (microgametogenesis) but does not affect sperm cell formation.

### DHQS deficiency does not affect pollen germination but significantly impairs pollen tube elongation in vitro

*In vitro* pollen germination and tube growth assays were performed to compare wild-type and *dhqs-1* mutant pollen. Both genotypes exhibited comparable pollen germination rates (~77%) when cultured on standard germination medium (Figure 1F, G). In contrast, quantitative analysis of pollen tube length revealed a significant impairment in tube elongation in the *dhqs-1* mutant after 4 h of culture. At this time point, wild-type pollen tubes had elongated to approximately twice the length of *dhqs-1* mutant tubes (Figure 1H). These results indicate that loss of DHQS function does not affect pollen germination competence, but specifically impairs the subsequent elongation phase of pollen tube growth.

### Shikimic acid modulates pollen-stigma interactions

To investigate the ability of *dhqs-1* mutant pollen tube growth on the stigma, we performed semi‑*in vivo* pollen tube growth assays. The results showed that only 1–2% of pollen tubes from *dhqs-1*/+ plants were *dhqs-1* mutant (GFP-positive) and successfully grew through the stigma, indicating that loss of DHQS function impairs the competence of pollen tube growth on the stigma during the critical early interaction with the stigma (Figure 1I). To determine whether this growth defect is caused by the absence of the DHQS itself or by the lack of its downstream product, shikimate, we supplemented the germination medium with exogenous shikimate. When cultured on medium containing 100 mg/L shikimate, 49% of pollen tubes from *dhqs-1*/+ plants were *dhqs-1* mutant (GFP-positive) and successfully grew through the stigma (Figure 1G). Furthermore, under these conditions, the length of the rescued GFP pollen tubes remained significantly shorter than that of wild‑type pollen tubes, with a reduction of approximately 30% (Figure 1K). These results demonstrate that growth defect in *dhqs-1* mutant pollen tubes can be rescued by exogenous shikimate supplementation.

Based on these observations, we conclude that gametophytic DHQS is dispensable for the formation of pollen grains and sperm cells. The interaction between pollen and the stigma involves a complex interplay of metabolic and signaling pathways, in which shikimate derived from pollen functions as a critical determinant for successful growth on the stigma. Pollen grains acquire the competence to grow on the stigma either by producing shikimate endogenously or by utilizing exogenous shikimate obtained from the external environment.

## Discussion

The proper interaction between pollen and the pistil is a cornerstone of successful fertilization in flowering plants.^20^ While the role of DHQS in female gametophyte development and pollen tube guidance has been previously established,^16^ its specific function in pollen development and the early stages of pollen-pistil interaction remained elusive. This study provides novel evidence that DHQS is a key regulator of pollen-pistil communication, specifically by mediating the ability of the pollen tube to grow through the stigma, an effect that is dependent on the synthesis of shikimic acid.

Our cytological analysis of the *dhqs-1*/+ mutant indicated that DHQS is dispensable for the fundamental processes of sperm cell specification and differentiation. However, the significant reduction in pollen grain size suggests that DHQS plays a role in pollen development, potentially by contributing to the accumulation of storage reserves or cell wall precursors during maturation, processes that often rely on primary metabolic pathways like the shikimic acid pathway.

The most striking finding of this study is the functional distinction between pollen germination *in vitro* and pollen tube behavior *in vivo*. The unimpaired germination rate of *dhqs-1* pollen on artificial medium confirms that the genetic lesion does not compromise the intrinsic ability of the pollen grain to hydrate and initiate tube growth. Typical in vitro assays could serve as an important direction for future research to further investigate the direct promoting effect of shikimic acid on dhqs-1 pollen tube growth. Conversely, the delayed growth observed in the semi-*in vitro* assay reveals a specific deficiency in the pollen's ability to navigate the physical and physiological barrier of the stigma. This decoupling of germination and growth is a common phenotype for mutants affected in pollen-pistil signaling, as the stigma actively controls the entry of pollen tubes into the style.

The significant alleviation of the *dhqs*-1 pollen tube growth defect by exogenous shikimic acid is particularly compelling. It provides direct metabolic evidence that the defect is linked to a deficiency in shikimic acid or its downstream aromatic metabolites. This suggests that the requirement of the pollen tube for shikimic acid-derived compounds becomes critical at the moment of stigma invasion. These compounds may serve as precursors for specialized metabolites needed to counteract stigma defenses, or they may be integral to the rapid remodeling of the pollen tube cell wall required to generate the necessary growth force. This finding positions the DHQS-mediated shikimic acid pathway as a crucial metabolic hub for pollen function during the critical pollen-stigma interaction.

Previous studies have highlighted the role of small signaling molecules and peptides, such as RALFs, in regulating this interaction.^3^ Our results add a new layer of complexity by demonstrating that primary metabolism- specifically the shikimic acid pathway within the pollen tube itself -is also a determinant of success in crossing the stigma. It is plausible that metabolic status and signaling pathways are integrated; for instance, signaling from the pistil (e.g., RALF peptides) might trigger a demand for rapid metabolic output in the pollen tube-a demand that cannot be met in the shikimic acid-deficient *dhqs-1* mutant.

In conclusion, this study reveals a previously unknown role for DHQS in regulating pollen-pistil interaction. It demonstrates that in addition to its function in female gametophyte development, DHQS is essential for optimal pollen tube growth through the stigma, an effect mediated by its role in shikimic acid production. These findings expand our understanding of the molecular and metabolic dialog between the male gametophyte and the pistil, identifying the shikimic acid pathway as a critical component of successful fertilization. Future research should focus on identifying the specific shikimic acid-derived metabolites that facilitate pollen tube growth and the potential downstream signaling pathways they influence.

## Data Availability

Data sharing is not applicable to this article as no new data were created or analyzed in this study.
